# Dipeptidyl peptidase-4 is increased in the abdominal aortic aneurysm vessel wall and is associated with aneurysm disease processes

**DOI:** 10.1371/journal.pone.0227889

**Published:** 2020-01-23

**Authors:** Moritz Lindquist Liljeqvist, Linnea Eriksson, Christina Villard, Mariette Lengquist, Malin Kronqvist, Rebecka Hultgren, Joy Roy

**Affiliations:** 1 Department of Molecular Medicine and Surgery, Karolinska Institutet, Stockholm, Sweden; 2 Department of Vascular Surgery, Karolinska University Hospital, Stockholm, Sweden; Max Delbruck Centrum fur Molekulare Medizin Berlin Buch, GERMANY

## Abstract

**Background:**

Abdominal aortic aneurysm (AAA) is a potentially life-threatening disease, and until today there is no other treatment available than surgical intervention. Dipeptidyl peptidase-4 (DPP4)-inhibitors, used clinically to treat type 2 diabetes, have in murine models been shown to attenuate aneurysm formation and decrease aortic wall matrix degradation, inflammation and apoptosis. Our aim was to investigate if DPP4 is present, active and differentially expressed in human AAA.

**Methods and results:**

DPP4 gene expression was elevated in both media and adventitia of AAA tissue compared with control tissue, as measured by microarrays and qPCR, with consistent findings in external data. The plasma activity of DPP4 was however lower in male patients with AAA compared with age- and gender-matched controls, independently of comorbidity or medication. Immunohistochemical double staining revealed co-localization of DPP4 with cells positive for CD68, CD4 and -8, CD20, and SMA. Gene set enrichment analysis demonstrated that expression of DPP4 in AAA tissue correlated with expression of biological processes related to B- and T-cells, extracellular matrix turnover, peptidase activity, oxidative stress and angiogenesis whereas it correlated negatively with muscle-/actin-related processes.

**Conclusion:**

DPP4 is upregulated in both media and adventitia of human AAA and correlates with aneurysm pathophysiological processes. These results support previous murine mechanistic studies and implicate DPP4 as a target in AAA disease.

## Introduction

Abdominal aortic aneurysm (AAA) is a mostly asymptomatic but life-threatening disease; the prevalence of AAA is approximately 1–2% in 65-year-old men and 0.5% in 70-year-old women [1]. Rupture of AAA, causing around 50% of those affected to die outside hospital and with a peri- and post-operative mortality of 30–40%, is the most feared complication [2,3]. To date, no pharmacological approach has been successful in preventing the growth or rupture of the aneurysm, leaving surgical intervention as the only therapy accessible for treating the disease [4]. Degradation of extracellular matrix (ECM) and elastin has been suggested to play an important role in the progression of the disease. Infiltrating inflammatory cells are a major source of proteases involved in the degradation of the aortic vessels wall, thus contributing to rupture [5]

Dipeptidyl peptidase-4 (DPP4 a.k.a. CD26) is a serine protease that exists as a membrane bound cell surface peptidase, and as a soluble form in the circulation [6]. DPP4 is expressed on a variety of cell types, such as T- and B-cells, natural killer cells, macrophages, epithelial and endothelial cells [7,8]. DPP4 targets many peptides, one of them being glucagon-like peptide-1 (GLP-1). GLP-1 is an incretin hormone that plays an important role in the regulation of glucose homeostasis and is used in clinical treatment of diabetes [9]. Over the years it has become evident that GLP-1 exerts extrapancreatic effects, so also in the cardiovascular system [10–12]. GLP-1 has a half-life of approximately two minutes before it is degraded by DPP4, thus complicating the application of native GLP-1 for treatment of diabetes [6]. Strategies to increase the presence of GLP-1 in the blood are used in the treatment of type 2 diabetes, either by using degradation resistant GLP-1 receptor agonists, or by inhibiting DPP4 activity.

Interestingly, favourable effects of both DPP4 inhibition and GLP-1 on AAA development and progression have been reported for animal models [13–18]. However, there are no reports showing the expression and activity of DPP4 in human aneurysm tissue. Our aim was therefore to investigate the role of DPP4 in human AAA disease.

## Materials and methods

### Tissue samples

Patients undergoing open elective surgery for AAA at our department were included into a prospective study and biobank on AAA pathophysiology, the Stockholm AAA Biobank (STAAAB), after giving informed consent. Clinical data was collected from patient records. Samples were excised from the thrombus-covered aspect of the anterior vessel wall, as most clinically significant AAAs feature an intraluminal thrombus [19]. Control samples were taken from the abdominal aorta of solid organ transplant donors. Samples for protein study were immediately frozen and subsequently stored in -80°C whereas those intended for RNA measurements were immersed in RNAlater (Invitrogen, ThermoFisher Scientific, Waltham, MA) and stored in +4°C for 48 hours, after which they were manually dissected into media and adventitia wall layers and stored in -80°C.

### Blood samples

Control blood samples were collected in ethylenediaminetetraacetic (EDTA) tubes from 65-year-old males without AAA who participated in the regional AAA screening program. Patient blood samples were collected pre-operatively in EDTA tubes from age-matched patients who underwent open surgical repair of their AAA at the Vascular Surgery department of the Karolinska University Hospital in Stockholm, Sweden. Platelet free plasma was prepared through an initial centrifugation of the blood samples at room temperature (20 min, 2500 *g*). The plasma obtained from the first centrifugation was then centrifuged again at 4°C (30 min, 20000 g) to remove the platelets. Samples were then stored at −80°C until further analysis.

### Dipeptidyl peptidase-4 activity assay

DPP4 activity was measured in human plasma using the DPP4 Activity Assay Kit (Sigma Aldrich, St. Louis, MO) according to the manufacturer’s instructions. The assay is based on the non-fluorescent substrate H-Gly-Pro-AMC that is cleaved by DPP4, thus releasing the fluorescent product 7-Amino-4-Methyl Coumarin which is detected by excitation/emission wavelength of 360/460 nm. This data is available in S1 Data.

### Isolation of RNA for real-time PCR and microarray analysis

For RNA isolation, tissue was put in RNAlater (Invitrogen, ThermoFisher Scientific, Waltham, MA) directly at surgery. Homogenization of tissue was performed using a TissueRuptor (Qiagen, Hilden, Germany) and the Qiazol Lysis Reagent (Qiagen, Hilden, Germany) with subsequent purification by RNeasy Mini kit or the miRNeasy Mini Kit (Qiagen, Hilden, Germany), including DNase digestion. The concentration and quality was measured using Nanodrop ND-1000 (ThermoFisher Scientific, Waltham, MA) and the quality estimated using a Bioanalyser capillary electrophoresis system (Agilent Technologies, Santa Clara, CA, USA).

### Real-time PCR of tissue samples

For qPCR, total RNA was reverse-transcribed using High Capacity RNA-to-cDNA kit (Applied Biosystems, ThermoFisher Scientific, Waltham, MA, USA). PCR amplification was done in 96-well plates in 7900 HT real-time PCR system (Applied Biosystems), using TaqMan^®^ Universal PCR Master Mix (Applied Biosystems) and TaqMan^®^ Gene Expression Assays (*DPP4*; Hs00897391_m, Applied Biosystems). All samples were measured in duplicates and results were normalized to the Ct values of *RPLP0* housekeeping control (Hs99999902_m1, Applied Biosystems).

### Immunohistochemistry

Immunohistochemistry of aortic tissue sections from AAA patients and organ donors was performed as previously described [20]. Antibodies targeted DPP4, CD20, CD4, CD8, CD68 and smooth muscle alpha actin (S1 Table). Representative images of stained sections were taken using a Nikon OPTIPHOT-2 microscope equipped with digital camera and NIS-Elements software.

### Microarray

Human Transcriptome Array 2.0 (Thermo Fisher Scientific) was used to analyze genome-wide mRNA expression of the samples at the Bioinformatics and Expression Analysis core facility at Karolinska Institutet in Stockholm, Sweden. The resulting CEL intensity files were normalized by Guanine Cytosine Count Normalization and Signal Space Transformation by use of the Transcriptome Analysis Console software (Thermo Fisher Scientific). All samples passed microarray quality control performed with the same software. DPP4 results are available in S1 Data.

### Statistical and bioinformatic analyses

Continuous data were expressed as median (interquartile range, IQR) and categorical data as number (percent). Differences between two groups were tested with Wilcoxon rank-sum test and Chi-squared test, respectively, for these data types. Correlations and associations were tested with Spearman’s rank correlation coefficient as well as linear uni-and multivariate regression. Any null hypothesis was rejected at p < 0.05. Analyses were performed with GraphPad Prism 8 (GraphPad Software Inc., San Diego, CA, USA) as well as R and R Studio programming language and software environment, with additional packages installed [21–25]. While two groups were primarily compared with Wilcoxon rank-sum test, the test of difference in the microarray results were also analysed by limma with age- and gender-adjustment and Benjamini-Hochberg consideration of multiple comparisons, with an accepted false detection rate (FDR) of < 0.05 [21]. Gene expression results were validated by analysis of data from two previously published microarray studies on AAA [26,27], using GEO2R on data available from Gene Expression Omnibus, accession numbers GSE7084 and GSE57691.

In order to investigate processes associated with DPP4 expression, all mRNAs from the microarray analysis was ranked according to their limma-derived log2 fold change [21] with DPP4 expression as the independent variable. The ranked list was input into gene set enrichment analysis (GSEA) [28] against gene ontology biological processes gene sets [29]. Significantly enriched gene sets were exported into Cytoscape [30], plotted into a network according to the overlap coefficient and manually summarized for the figure. The Genotype-Tissue Expression (GTEx) database was queried for normal tissue and cell expression of DPP4 [31].

### Ethics statement

Patients gave informed consent to blood- and tissue-sampling preoperatively. Controls gave informed consent to blood sampling at the time of screening and organ donors consented to the use of tissue for research purposes at the time of enlisting to the donation registry. The study was approved by the regional ethics review board in Stockholm, registration numbers 00–337 (KI Forskningsetikkommitté Nord), 2009/1098-32, 2009/09-31/4 and 2013/615-31/4 (Regionala Etikprövningsnämnden i Stockholm).

## Results

### Clinical characteristics of study subjects

The clinical characteristics of screening controls and patients with AAA are presented in Table 1. The patients included into the microarray analysis were significantly older than the controls, and there was a trend towards a smaller proportion of women. Patients and controls from whom plasma samples were collected did not differ in age or BMI. However, this patient group consisted of significantly more smokers and also displayed a higher prevalence of cardiovascular disease, hypertension and more diabetes type 2, with corresponding differences in the prescribed drugs.

**Table 1:** Clinical characteristics of control subjects and aneurysm patients.

	Tissue samples Microarray analysis			Plasma samples DPP-4 activity		
	AAA n = 76	Controls n = 13	P	AAA n = 49	Controls n = 42	P
**Age (years)**	69 (65–75)	53 (44–68)	0.0007	65 (63–67)	65 (-)	-
**Gender (men)**	60 (79%)	7 (54%)	0.11	49 (100%)	42 (100%)	-
**Smoking (current/previous or never)**	31/45 (41/59%)	3/10 (23/77%)	0.37	22/24/3 (45/50/6%)	6/15/21 (14/35/50%)	5.2e-06
**Aneurysm Diameter at surgery (mm)**	60 (56–70)	NA	-	-	-	-
**Diabetes (any type)**	5 (7%)	NA	-	12 (24%)	2 (5%)	0.024
**BMI (kg/m**^**2**^**)**	25 (23–28)	NA	-	27 (25–30)	26 (24–28)	0.17
**Hypertension**	55 (72%)	NA	-	37 (76%)	17 (40%)	0.0015
**Previous myocardial infarction**	17 (22%)	NA	-	16 (33%)	3 (7%)	0.0064
**Aspirin**	47 (62%)	NA	-	36 (73%)	4 (10%)	5.1e-09
**ACE-inhibitors**	20 (26%)	NA	-	15 (31%)	5 (12%)	0.075
**Angiotensin II receptor blockers**	14 (18%)	NA	-	13 (27%)	5 (12%)	0.17
**Beta-blockers**	29 (38%)	NA	-	23 (47%)	5 (12%)	0.0012
**Statins**	55 (72%)	NA	-	23 (47%)	5 (12%)	0.0012
**Insulin**	0 (0%)	NA	-	0 (0%)	1 (2%)	0.94
**Any oral anti-DM**	3 (3.9%)	NA	-	8 (16%)	1 (2%)	0.062

Continuous data are described as medians (interquartile range) and categorical data as numbers (percent). A p-value below 0.05 was considered significant using the Chi-squared test or Wilcoxon rank-sum test. Missing data are described as numbers of patients with missing data (percent).

Abbreviations: AAA; abdominal aortic aneurysm, BMI—Body Mass Index, here presented as median values, IQR–inter quartile range

### DPP4 is upregulated in abdominal aortic aneurysms

**Fig 1:**
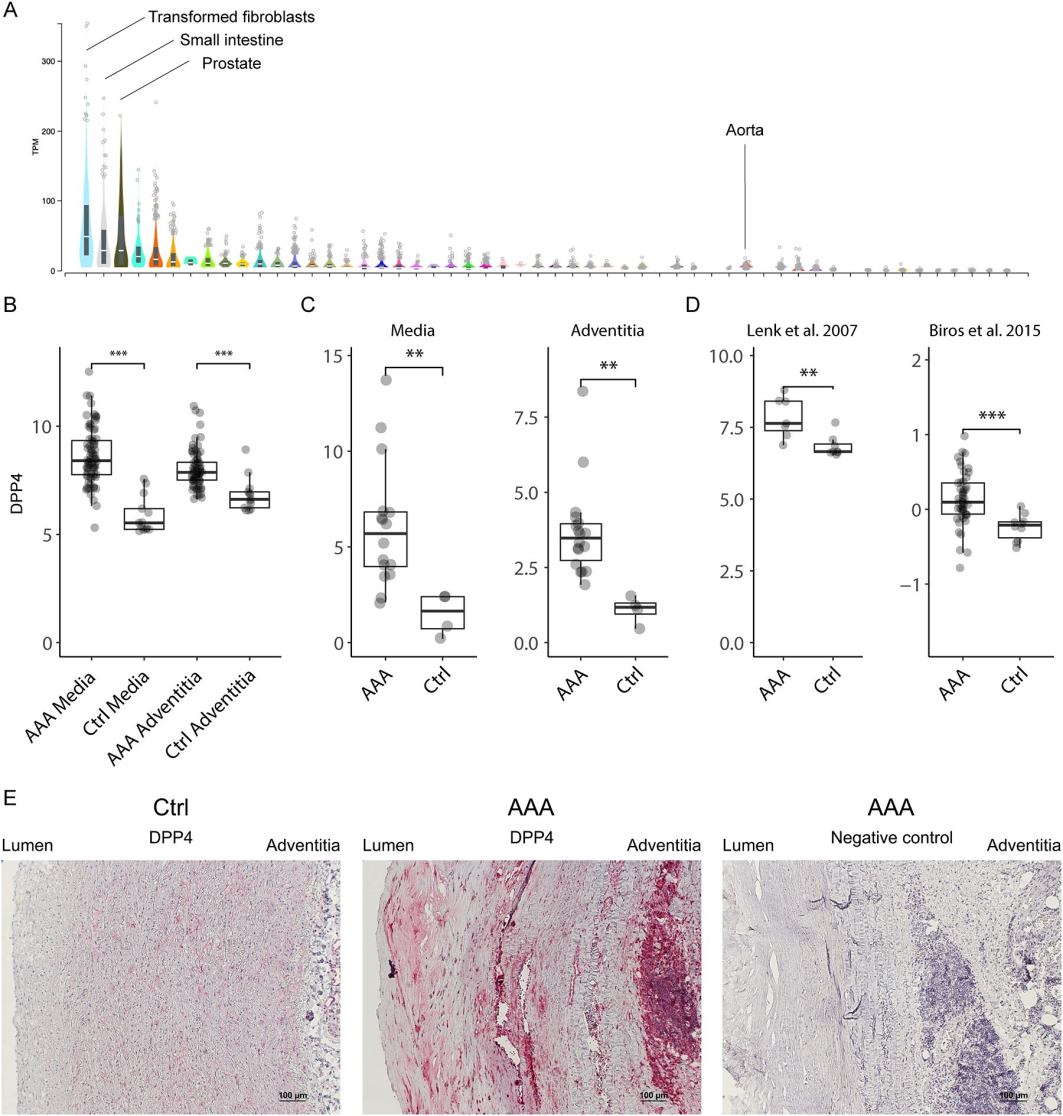
DPP4 expression in normal aorta and AAA. A: Normal DPP4 gene expression in different tissues, data from the genotype-tissue expression (GTEx) database, in which genome-wide gene expression is available from different disease-free tissues of hundreds of human donors. B and C: Gene expression of DPP4 as measured by microarray (B) and qPCR (C). D: Analysis of DPP4 gene expression data from two previously published microarray studies. E: Protein expression of DPP4 was analyzed with immunohistochemistry. Abbreviations: AAA; abdominal aortic aneurysm, Ctrl; controls. *; p<0.05, **; p<0.01, ***; p<0.001.

The tissue and cell expression of DPP4 was studied in the GTEx online resource which contains gene expression data of different human organs/tissues in normal states, as well as in several cell lines (Fig 1A). Expression of DPP4 in the normal aorta was low. The highest expression of DPP4 was seen in transformed fibroblasts, followed by terminal ileum, prostate, kidney cortex, adipose tissue, lung and spleen.

Microarray analysis of the AAA wall in 76 patients and 13 controls showed a significant increase in DPP4 expression in both media and the adventitia (Fig 1B), which persisted after limma adjustment for age, gender, smoking and FDR-adjustment for multiple comparisons (not shown). The upregulation of DPP4 was validated with qPCR (Fig 1C). We further analysed data from two previously published microarray studies that compared full-thickness vessel wall of AAA with that of control aortas [26,27]. The above findings were substantiated in these external data, with a DPP4 fold change of 2 (p = 0.0059) and 1.3 (p = 0.00015), respectively (Fig 1D). To confirm the presence of the DPP4 protein in the aortic wall, immunohistochemical staining was performed. There was a strong expression of DPP4 in most cells throughout the AAA vessel wall, including those present in inflammatory infiltrates, neovessels and aneurysm media (Fig 1E). DPP4 was also detected in control aortas, however the DPP4 staining in the vessel wall of AAA patients was qualitatively stronger.

### DPP4 activity is lower in plasma of AAA patients compared with screening controls

**Fig 2:**
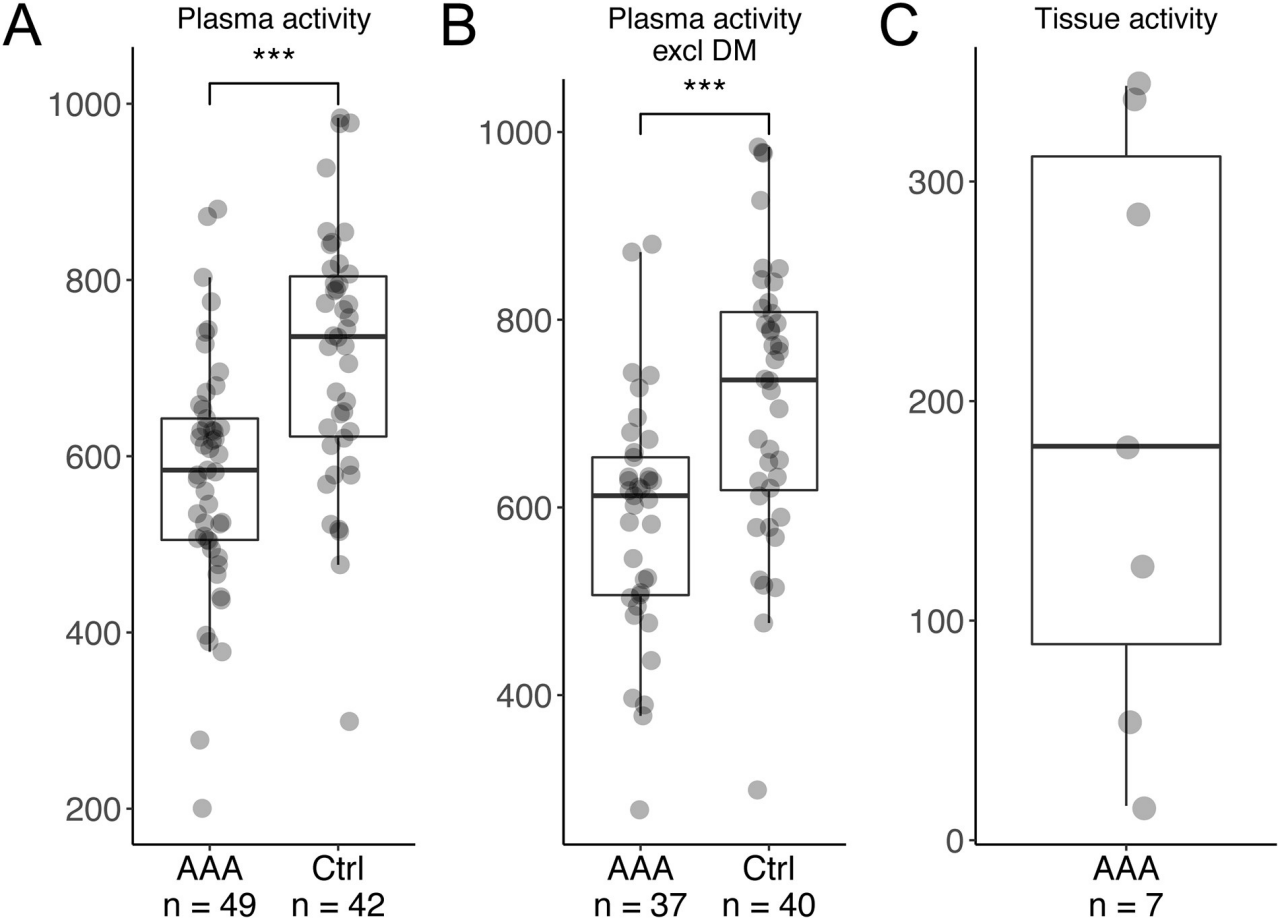
DPP4 activity in plasma. A and B: DPP4 activity in plasma from patients with AAA compared with age-matched controls; with diabetic patients and controls included (A) and excluded (B). C: DPP4 activity in the thrombus-covered wall. Abbreviations: AAA; abdominal aortic aneurysm, Ctrl; controls, DM: diabetes mellitus. *; p<0.05, **; p<0.01, ***; p<0.001.

The activity of DPP4 was measured in plasma sampled preoperatively in patients undergoing surgery of their AAA and in 65-year-old male controls attending screening for AAA. DPP4 activity in plasma of the AAA patients was lower compared to the controls (Fig 2A). Similar results were observed when only non-diabetic subjects were included (Fig 2B). In multivariate regression, DPP4 activity was negatively associated with diagnosis of AAA and positively with active smoking (Table 2). No correlation was observed between the aneurysm maximal diameter and the plasma DPP4 activity (r = -0.12, p = 0.42). To determine whether DPP4 was also active and not only expressed in the vessel wall, its activity was measured in tissue samples taken from the aneurysm wall of 7 patients undergoing open surgery. Although the activity varied between the samples, we were able to detect DPP4 activity in the vessel wall (Fig 2C).

**Table 2:** DPP4 activity and patient characteristics.

Dependent	Independent	Coefficient	P value
	Univariate regression		
**DPP4 activity in plasma**	AAA	-159	1.5e-06***
	Active smoking	55.3	1.2e-01
	Angina, history of	-31.6	6.2e-01
	AMI, history of	-66.1	1.5e-01
	Hypertension	-36.1	3.1e-01
	Diabetes type 2	-117.2	1.7e-02*
	Aspirin	-88.1	1.4e-02*
	Statins	-63.8	7.2e-02
	Betablockers	-68	8.4e-02
	ACE Inhibitor	-32.7	4.4e-01
	Calcium channel blocker	-24.9	5.4e-01
	Angiotensin II receptor blocker	-4.1	9.3e-01
	**Multivariate regression**		
	AAA	-174	2.9e-04***
	Active smoking	74.9	1.8e-02*
	Diabetes type 2	-61.2	2.0e-01
	Aspirin	1.7	9.7e-01
	Statins	39	3.6e-01
	Beta blockers	-9.8	7.9e-01

Univariate linear regression of patient characteristics as independent and DPP4 plasma activity as dependent variable and multivariate linear regression on significant results and trends.

Abbreviations; DPP4; dipeptidyl peptidase 4, AAA: abdominal aortic aneurysm, ACE: angiotensin converting enzyme, AMI: acute myocardial infarction.

*: p < 0.05,

***: p < 0.001.

### DPP4 co-localizes with infiltrating inflammatory cells and smooth muscle cells in the aneurysm vessel wall

**Fig 3:**
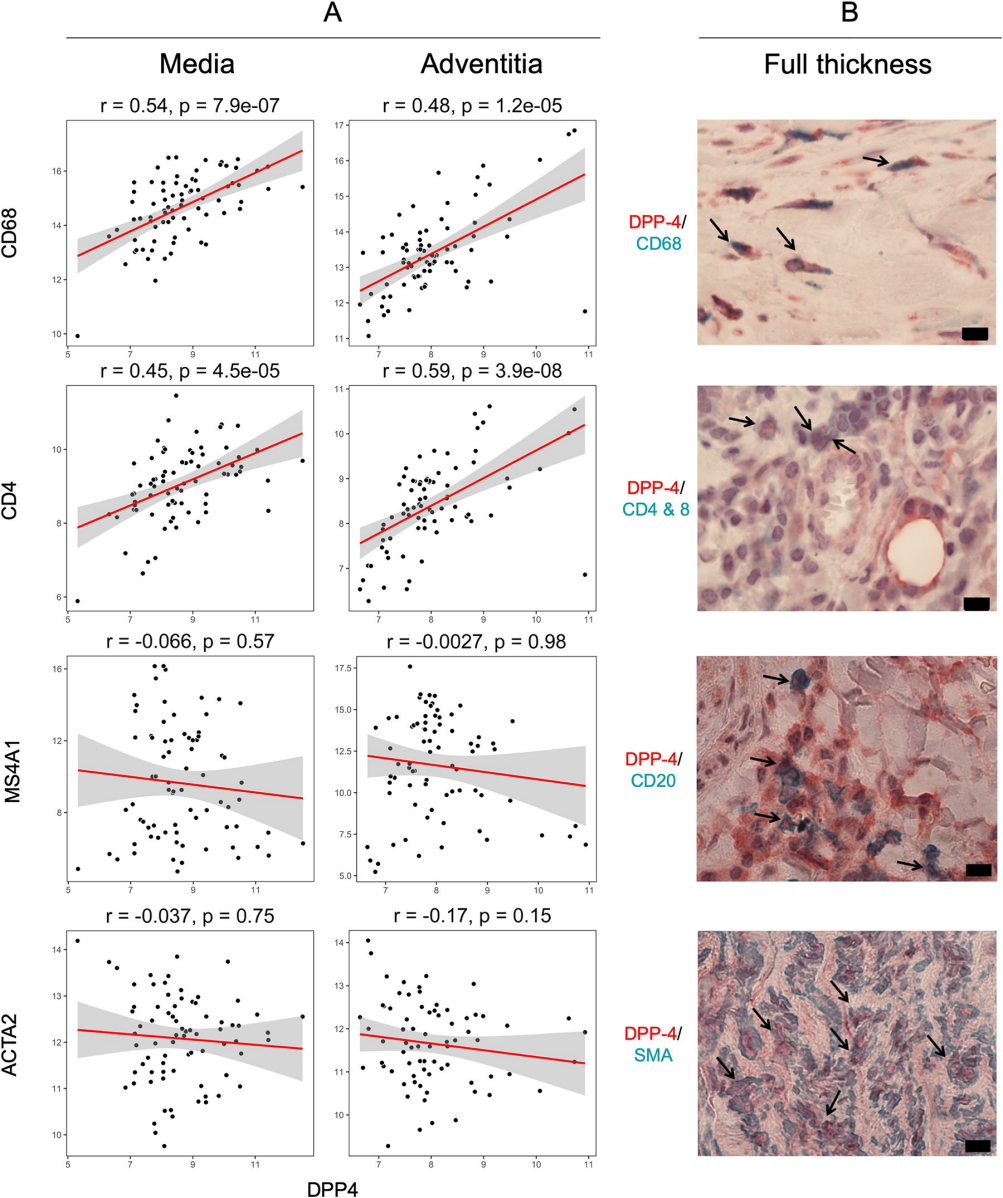
Correlation and co-localization of DPP4 gene and protein with leukocyte and smooth muscle cell-markers in the aneurysm wall. A: Spearman correlation between the expression of DPP4 and CD68, CD4, MS4A1 and ACTA2, as approximate markers for macrophages, CD4+ T-cells, B-cells and smooth muscle cells. B: Immunohistochemichal double staining to between DPP4 and CD68, CD4&8, CD20 and SMA as markers for above cell types. Arrows indicate co-localization.

Additional analysis from microarray data showed that DPP4 correlated with the expression of the macrophage marker CD68 and the T-cell marker CD4 both in the media and the adventitia (Fig 3A). In contrast, there was no correlation between mRNA expression of DPP4 and the B-cell marker CD20/MS4A1 or smooth muscle cell alpha actin / ACTA2. There were negative correlations between the gene expression of DPP4 and smooth muscle cell (SMC) markers MYH11 (media: r = -0.26, p = 0.024, adventitia: r = -0.32, p = 0.0016) and SMTN (media: r = -0.36, p = 0.0014, adventitia: r = -0.36, p = 0.0016). Immunohistochemical staining of the AAA vessel wall demonstrated that DPP4 co-localizes with macrophages and T-cells. Despite the absence of a positive association in the microarray data, DPP4 co-localized with B-cells and SMCs in the aneurysm wall (Fig 3B).

### DPP4 correlates with increased expression of inflammation-, proteolysis- and apoptosis-related genes and a decreased expression of those related to muscle cells

**Fig 4:**
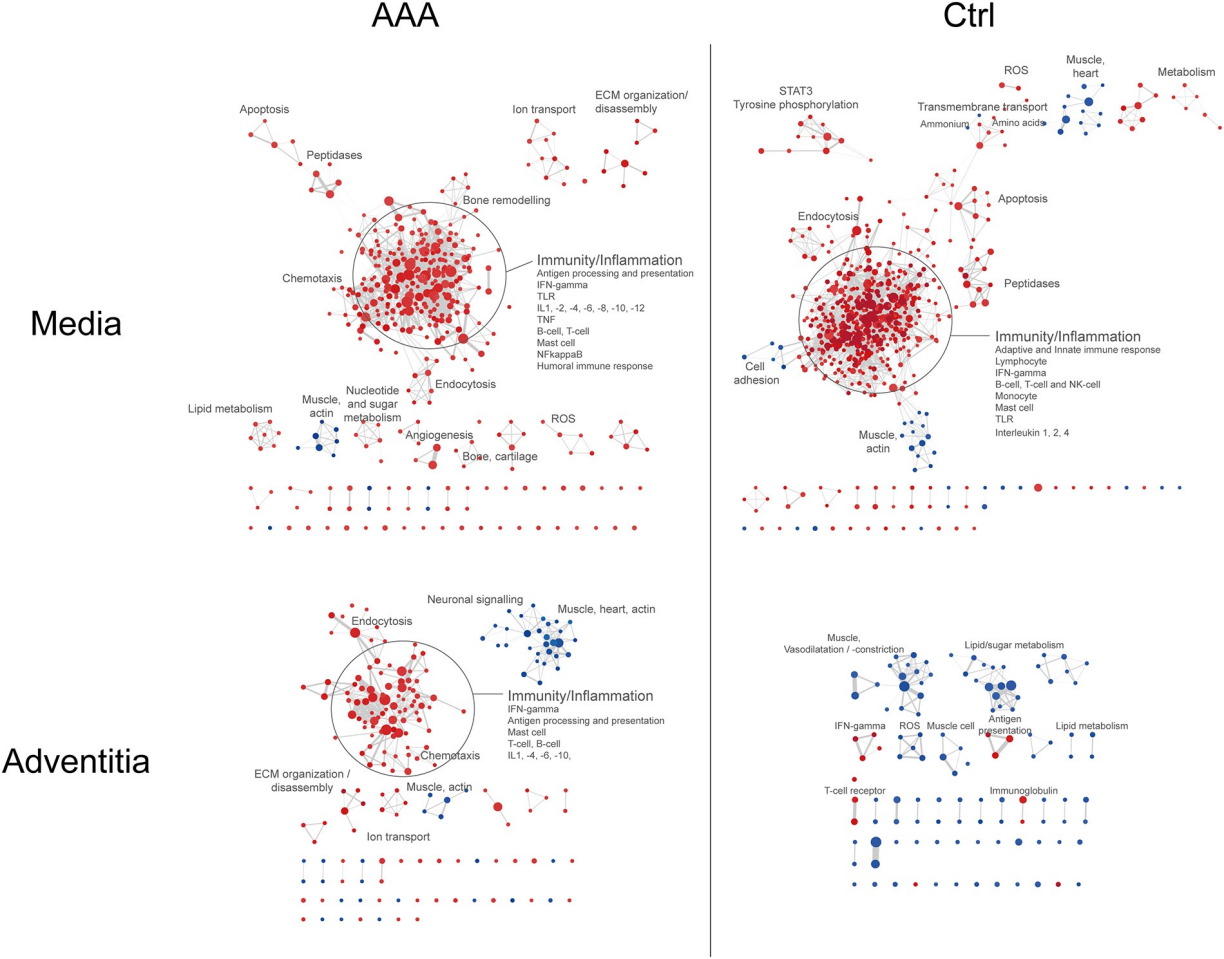
Gene set enrichment analysis of genes co-expressed with DPP4. Genes were ranked according to association with DPP4 expression, as calculated by the limma package in R, and input into GSEA. Networks of enriched biological processes were created in Cytoscape. Each node represents a significantly enriched biological process, where red color represents upregulation, blue color downregulation and the point size corresponds to the number of genes included into the set. Edges correspond to the number of genes in common between two nodes, normalized against the size of the smallest gene set. Abbreviations; ECM: extracellular matrix, ROS: reactive oxygen species.

The expression of DPP4 in aneurysm and control media correlated with sets of mRNAs associated with numerous immune processes such as migration/chemotaxis of granulocytes, monocytes and lymphocytes, antigen presentation and those related to T- and B-cells, mast cells, macrophages, as well as extracellular matrix-, peptidase-, apoptosis-, metabolism- and oxidative stress-associated gene sets (Fig 4). However, the positive association between DPP4 and angiogenesis was unique to the aneurysm media. An inverse association between DPP4 and muscle-/actin-related processes was seen in both AAA and control media.

In the adventitia of AAA and controls, DPP4 was similarly associated with a smaller number of immune processes and inversely related to muscle/actin processes. The aneurysm adventitia showed a positive association between DPP4 expression and extracellular matrix processes, such as extracellular matrix disassembly and collagen fibril organization, whereas for the control adventitia, there was only a negative association between extracellular matrix assembly and DPP4. Further, some neuronal and several specific immune-related processes were inversely and directly associated with DPP4, respectively, specifically in the aneurysm. In the control adventitia, DPP4 was inversely related to processes involving vasoconstriction/-dilatation, which was not seen in the aneurysm adventitia.

## Discussion

The current study showed that gene expression of DPP4 is transmurally increased in the AAA vessel wall compared with control aortas from organ donors. The gene expression of DPP4 in AAA media and adventitia correlated with genes related to typical aneurysm disease processes, such as a large number of immune processes, ECM degradation and peptidase activity, angiogenesis and reactive oxygen species, whereas there was an inverse association with processes typically downregulated in aneurysm disease, including those related to SMCs. In GTEx, there was very low DPP4 expression in normal aorta and high expression in transformed fibroblasts. The DPP4 protein was active in the AAA vessel wall and co-localized with cells expressing macrophage-, T-cell-, B-cell- and SMC-markers. However, the DPP4 activity was lower in plasma from patients with AAA compared with control subjects, independently of diabetes and medications but with indications of increased activity with smoking.

Both microarray analysis and qPCR validation, as well as examination of data from previously published microarray studies, revealed an increased expression of DPP4 in the media, adventitia and full-thickness wall of the AAA compared to donor aortas. The DPP4 expression also significantly correlated with markers of macrophages and T-cells. The reason why an increased expression of DPP4 in the aneurysm wall was observed might be a reflection of the infiltrating inflammatory cells, which is one of the hallmarks of the disease [32]. Also, soluble DPP4 increases the inflammatory response in SMC through activation of NF-kB signalling. In turn, this leads to an increased production and secretion of inflammatory cytokines and iNOS activation [33], thus adding to the inflammation in the vessel wall. However, SMCs themselves have also been shown to produce and shed DPP4 upon hypoxia [34] and since the thrombus-covered wall is hypoxic [35,36], DPP4 produced by SMCs probably also contribute to the increased expression that we detected. Protein DPP4 co-localized with SMCs upon immunohistochemistry but there was no positive correlation of gene expression between SMC markers and gene DPP4. This could at least in part be explained by a decreasing amount of SMCs in AAA with increasing inflammation, thus would the total relative SMC-related mRNA concentration decrease, even as SMCs do appear to express DPP4 in the aneurysm. However, inhibitors of DPP4 have been shown in other studies to decrease SMC proliferation [37–39], resulting in decreased neointima formation and atherosclerosis. While SMC apoptosis is a strong feature of AAA, the role of SMC proliferation and phenotypic modulation in AAA remains to be fully elucidated.

On the other hand, the current study detected a decreased activity of DPP4 in plasma compared to control subjects without AAA, independent of comorbidity and medication. While an increased activity does not equal increased expression, a speculative explanation could be recruitment of inflammatory cells into the aneurysm wall, thus decreasing their presence in plasma and resulting in a lower DPP4 activity when measured. The decreased activity of DPP4 in plasma is congruent with some studies, while it stands in contrast to others. Krizhanovskii et al showed increased GLP1 in plasma of patients with dilatation of the ascending aorta, which could imply a decreased DPP-4 activity, and other studies have found a decreased DPP-4 activity in the plasma of patients with chronic obstructive pulmonary disease, a disease that has been linked to AAA rupture [40–42]. However, Lu et al detected a significant increase in DPP-4 in plasma from patients with AAA compared with the control group [16]. This discrepancy cannot be fully explained but our groups of study subjects differ, where Lu et al had a younger group of controls compared to the AAA patients. Our groups were age matched, and since it is unclear how age affects the DPP4 activity, this might be a reason for our differing findings. However, a limitation of the current study were the different settings in which blood from controls (at a screening centre) and patients (pre-operatively at admission) was drawn.

To further investigate what processes DPP4 gene expression is associated with in control and aneurysm vessel wall, GSEA of genes that showed a positive or inverse relationship to the expression of DPP4 was performed. It was observed that DPP4 expression correlated with typical aneurysm disease processes, such as a high degree of inflammation, ECM degradation and organization and peptidase activity [43]. There was downregulation of muscle cell-related processes, also a hallmark of aneurysm disease [43]. The positive association between DPP4 and angiogenesis, the latter being a result of a hypoxic environment under the intraluminal thrombus and also found to be increased at the site of rupture in AAAs, was unique to the AAA media [44].

Animal studies have suggested that inhibitors of DPP4 are protective against aneurysm development and suppresses many typical aneurysm disease processes [18]. In a rat model it was associated with a reduction of oxidative stress as well as reduced MMP2 and -9 expression, when administered before the induction of the aneurysm [13]. In another study, DPP4 inhibition decreased the formation of aneurysm, elastin degradation, expression of MMP2 and -9, gelatinolytic activity, aortic apoptotic signal and macrophage infiltration in a mouse model, with similar results shown for GLP-1 [14]. All animal model have different limitations and studying DPP4 inhibition in other recently developed models could yield additional insight [45,46]. Further, DPP4 is known to interact with fibronectin and collagen [47,48], and has been shown to be involved in a complex with fibroblast activation protein α that locally degraded ECM thus enabling endothelial cell invasion and migration [49]. DPP4 is also known to form a complex with adenosine deaminase (ADA). Upon ADA binding plasminogen-2 is activated, causing a degradation of ECM proteins such as collagen IV, fibronectin and proteoglycans. Plasminogen-2 activation can also lead to activation of MMPs, suggesting that DPP4 and ADA are involved in tissue remodelling [50,51]. Collectively, these preclinical results suggest a pathophysiologic mechanism of DPP4 in AAA and are congruent with the bioinformatic human data presented in the current study.

The current study is the first to examine the expression and role of DPP4 in human AAA. However, some limitations merit consideration. Due to a scarcity of donor tissue and few samples with sufficient amounts of total protein, the expression and activity in AAA tissue was verified by immunohistochemistry and activity assay, the latter prioritized above western blot experiments. Further, the herein presented data is observational and cross-sectional, which means that it should be interpreted in light of previously performed mechanistic studies.

## Conclusions

Animal studies have shown that treatment with DPP4 inhibitors can suppress the development and the progression of experimental aneurysms. We showed an increased medial and adventitial gene expression of DPP4 in AAA compared with controls, and demonstrated DPP4 activity in the human aneurysm vessel wall. DPP4 gene expression further correlated with the expression of genes related to typical AAA processes and the protein was expressed by macrophages, T-cells, B-cells and SMCs in aneurysm tissue. Together, previous experimental literature and the herein presented human data support the notion of DPP4 inhibition as a potential pharmaceutical intervention to be tried in AAA.

## Supporting information

S1 Table Antibodies used for immunohistochemistry. (DOCX)

S1 Data Supplemental data. (ZIP)
