# Supplementary material for: Dipeptidyl peptidase-4 is increased in the abdominal aortic aneurysm vessel wall and is associated with aneurysm disease processes
Source: PLoS One. 2020 Jan 23;15(1):e0227889. doi: 10.1371/journal.pone.0227889 (PMC6977716; doi:10.1371/journal.pone.0227889)
Supplement: S1 Table — (DOCX) [file pone.0227889.s001.docx]

**Supplemental table: Antibodies used for immunohistochemistry**

| **Antibody** | **Company** | **Cat. Nr** | **Dilution** | **Host** |
| --- | --- | --- | --- | --- |
| DPP4 | Abcam | ab28340 | 1/200 | Rabbit |
| CD68 | Novocastra | ncl-l-cd68 | 1/50 | Mouse |
| CD20 | Neomarker | ms-240-s1 | 1/100 | Mouse |
| CD4 | Novocastra | ncl-l-cd4368 | 1/50 | Mouse |
| CD8 | Novocastra | ncl-l-cd8295 | 1/50 | Mouse |
| SMA | DAKO | M0851 | 1/500 | Mouse |
